# Expanding the phenotype of the recurrent truncating eIF2γ pathogenic variant p.(Ile465Serfs*4) identified in two brothers with MEHMO syndrome

**DOI:** 10.1002/ccr3.5989

**Published:** 2022-06-19

**Authors:** Sofia Ygberg, Anna Lindstrand

**Affiliations:** ^1^ Neuropediatric Unit Karolinska University Hospital Stockholm Sweden; ^2^ Department of Clinical Genetics and Centre for Inherited Metabolic Diseases (CMMS) Karolinska University Hospital Stockholm Sweden; ^3^ Department of Molecular Medicine and Surgery Karolinska Institutet Stockholm Sweden

**Keywords:** EIF2S3, MEHMO, X‐linked genetic disease

## Abstract

We describe two brothers with a recurrent truncating *EIF2S3* variant and MEHMO (Mental retardation, Epileptic seizures, Hypogonadism and ‐genitalism, Microcephaly, Obesity). Both had the previously described facial dysmorphic features, microcephaly, developmental impairment, hypoglycemia, hypothyreosis, diabetes mellitus, epilepsy, hypertonus, obesity, and micropenis. Additionally, we describe hypothermia and reduced umbilical blood flow.

## Graphical abstract

MEHMO is a rare X‐linked genetic disease in boys with distinct dysmorphic features, severe developmental delay, epilepsy, endocrinological deficits, and hypothermia. MEHMO syndrome is caused by mutations in *EIF2S3* and can be inherited from an unaffected mother or arise de novo.

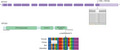

## 1 INTRODUCTION

There are many genetic conditions that are linked to various aspects of protein translation in humans, including Vanishing White Matter Disease and Wolcott–Rallison syndrome. The main regulatory step of translation is the initiation phase. To initiate protein translation in a mammalian cell, a ternary complex consisting of one GTP, the initiator methionyl‐tRNA, and the translation initiation factor eIF2 is required. The core subunit is called eIF2γ, and is encoded by *EIF2S3*, located on the X‐chromosome. The first paper linking this gene to disease appeared in 2012, when a missense variant was linked to a clinical phenotype with moderate‐to‐severe ID, microcephaly, short stature, and facial dysmorphic features in three male patients of the same family.^1^ Subsequently, a more severe phenotype with severe developmental delay, hypertonus, epilepsy, and short lifespan was described to result from a truncating variant in *EIF2S3*.^2^ A genetic retrospective analysis of a clinical group of patients described in 1998 as suffering from MEHMO syndrome (MIM# 300148)^3^ revealed that this group had pathogenic variants in *EIF2S3*. The first description included **M**ental retardation, **E**pileptic seizures, **H**ypogonadism and genitalism, **M**icrocephaly, **O**besity, and hence the acronym. Here, we describe an expanded MEHMO phenotype in two brothers with attenuating variants in *EIF2S3*.

### 1.1 Key features

Microcephaly, Hypoglycemia, Diabetes Mellitus, Hypothyreosis, Epilepsy, Hypertonus, Obesity, Micropenis

## 2 MATERIAL AND METHODS

Ethics approval was given by the Regional Ethical Review Board in Stockholm, Sweden (ethics permit number 2012/2106‐31/4). Written consent from the family was provided for genetic studies and for the publication of the cases including pictures.

Whole exome sequencing was performed and analyzed as previously described.^4^

## 3 CLINICAL CASE AND RESULTS

**FIGURE 1:**
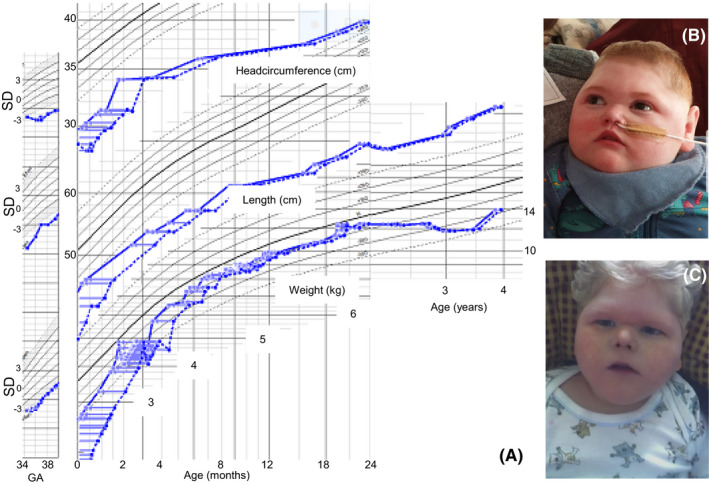
(A) Growth chart showing head circumference, weight, and length, in relation to standard curves from sibling born 2015. (B) Picture of sibling born 2015. (C) Picture of sibling born 2001

The index case was the third child of non‐consanguineous parents. He was delivered prematurely with C‐section at gestational age 34 + 4 due to reduced umbilical blood flow. It was noted that the umbilical cord was thick and had a jelly‐like appearance. His weight was 1627 g (‐3SD), his head circumference 28.5 cm (‐3SD) and his length 39 cm (‐4SD) (Figure 1A). His APGAR was 9,10,10, and apart from an early hypoglycemic event, and one suspected seizure, he was stable during the neonatal period. Magnetic resonance imaging (MRI) performed at 2 months age showed large ventricles with thin periventricular white matter, an underdeveloped corpus callosum, and a delayed myelination. Metabolic investigation did not reveal any metabolic cause. CGH‐array did not show any alterations of significance. He initially had repeated episodes of hypoglycemia and was diagnosed with hyperinsulinemia, for which he received diazoxide. This induced a lung edema and was discontinued. He subsequently developed an insulin dependent diabetes, for which he received insulin. He also had a poor exocrine pancreatic function, with low pancreatic amylase, despite normal ultrasound of the abdomen, and required substitution of fat‐soluble vitamins. He did, however, never develop pancreatitis. Additionally, he had numerous central endocrinologic symptoms, including hypothyreosis, obesity, and micropenis. At age 3 months, he developed treatment refractory epilepsy with multiple seizure types including generalized tonic–clonic, migrating focal, myoclonias, and absences. The treatment included combinations of levetiracetam, lamictal, lakosamide, clonazepam, fenobarbital, and zonisamid. In addition, he was often hypothermic, even at ambient room temperature, down to 32° rectal temperature. He had until his death, in multiorgan failure, virtually no development. He had a congenital microcephaly with progression overtime, and obesity, despite adequate nutrition (Figure 1A,B).

**FIGURE 2:**
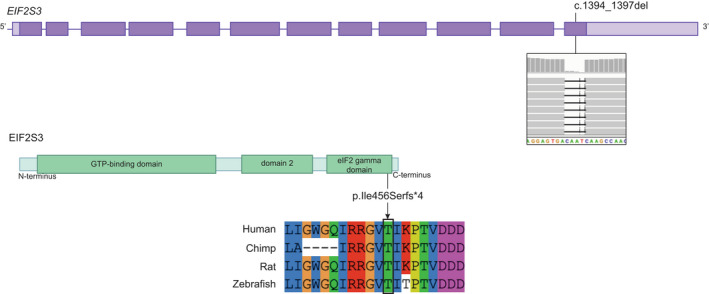
On top, a schematic drawing of the eIF2γ protein. The location of the variant in the 3′ end is indicated with a black line and a screen shot from the Integrative Genomics Viewer (IGV) illustrating the 4 bp deletion. Below the EIF2S3 protein structure is outlined with the variant indicated as well as conservation in four different species

He also had an older brother (Figure 1C), born in Germany 2001, with an almost identical clinical presentation, with the exception of the hypothyreosis. He also died at age 4 years. Whole exome sequencing revealed a hemizygous four basepair deletion in EIF2S3, c.1394_1397del, located on the X‐chromosome in both boys and their mother who was a healthy heterozygous carrier of the variant. The variant leads to a premature stop codon p.(Ile465Serfs*4) in the final exon of the gene (Figure 2).

## 4 CONCLUSIONS

We describe two boys with a clinical picture that included premature birth due to reduced umbilical blood flow, growth retardation, congenital microcephaly, hypothermia, micropenis, hypothyreosis, obesity, diabetes, and exocrine pancreatic failure. We found that both boys carried a truncating pathogenic variant towards the C‐terminal end of eIF2γ, c.1394_1397del p.(Ile465Serfs*4). This is the exact same variant described in the paper by Moortgat et al.^2^ That family was of Spanish origin and the mother in our family originates from northern Sweden, and we do not suspect a common heritage indicating that the variant has arisen independently at least twice. There are several similarities between the affected individuals in Family 2 described by Moortgat et al.^2^ and the cases reported here; including characteristic facial dysmorphic features (Figure 1B,C), poor development, severe congenital microcephaly (‐3SD vs. ‐4SD), initial hypoglycemia, epilepsy, hypertonia, micropenis, and finally death due to multiorgan failure. In contrast, our patients survived longer (4 years vs. 12 months), which may partially explain the additional phenotypes. Some phenotypes were known from other MEHMO cases, such as diabetes, poor pancreas function, and obesity.^1^, ^3^, ^5^, ^6^, ^7^, ^8^ However, neither the hypothermia, nor the reduced umbilical blood flow, that resulted in premature delivery in our cases were previously described.

Human body temperature is sensed by a system of thermoreceptors located in the core and the periphery, in order to detect real and anticipated temperature alternations, respectively. Signals from these thermoreceptors are connected to the lateral parabrachial nucleus (LBP) of the pons and the pre‐optic area (POA) located just by the anterior hypothalamus. Signals indicating low temperature will activate physiological responses such as vasoconstriction, shivering, and non‐shivering brown adipose thermogenesis. Structural damage to the LBP is known to abolishes behavioral temperature responses.^9^ POA is key to activation of all the physiological responses. Our patients had signs of vasoconstriction with colder extremities, though they were never seen shivering, indicating a partial failure to activate compensating systems. In addition, the anterior hypothalamus is important for thyroid regulation, growth hormone, and gonadotropic hormones, all affected in our boys. Structural defects in the hypothalamus were not detected in our patients, but the MRI investigations were not optimized to study the hypothalamus.

The ultrasounds performed during pregnancy indicated initial normal fetal growth, but in mid pregnancy (week 31), reduced fetal growth was noted. Both boys were delivered by C‐section at 34 + 4 and 35 + 3, respectively, due to absent umbilical artery end diastolic flow, indicating primary increased placental resistance or secondary such due to fetal hypoxia. Neither the MRI performed, nor the clinical picture, indicated chronic or acute hypoxia. The molecular cause remains elusive.

The two novel phenotypes, hypothermia and reduced umbilical flow has, to our knowledge, not been associated with any other conditions with affected translation initiation such as Vanishing White Matter disease (MIM# 606686, MIM# 606454, MIM# 606273, MIM# 606687, MIM# 603945; *EIF2B1*, *EIF2B2*, *EIF2B3*, *EIF2B4* and *EIF2B5*), variants in PPP1R15B, Wolcott Rallisson syndrome (MIM# 226980; EIF2AK3), or MEDS syndrome (MIM# 614231; IER3IP1). A recent paper has unraveled the exact molecular mechanism of this specific variant.^10^ These authors show that the variant impairs eIF2 function and leads to a chronic activation of the integrated stress response and neuronal differentiation results. Intriguingly, this can be reversed by the small molecule ISRIB, which raises treatment hopes in this devasting disease.

## AUTHOR CONTRIBUTIONS

SY wrote the manuscript and summarized clinical information. AL edited manuscript and analyzed genetic data.

## CONFLICT OF INTEREST

AL received honoraria from Illumina.

## CONSENT

Written informed consent was obtained from the patient to publish this report in accordance with the journal's patient consent policy.

## Data Availability

All relevant data is presented within the articel text. The whole genome sequencing data in this study is not publicly available due to privacy or ethical restrictions.
